# Meshing complex macro-scale objects into self-assembling bricks

**DOI:** 10.1038/srep12257

**Published:** 2015-07-30

**Authors:** Adar Hacohen, Iddo Hanniel, Yasha Nikulshin, Shuki Wolfus, Almogit Abu-Horowitz, Ido Bachelet

**Affiliations:** 1 Faculty of Life Sciences and the Institute of Nanotechnology & Advanced Materials, Bar-Ilan University, Ramat Gan, Israel; 2 Faculty of Mechanical Engineering, Technion Israel Institute of Technology, Haifa, Israel; 3 Department of Physics, Faculty of Exact Sciences, Bar-Ilan University, Ramat Gan, Israel

## Abstract

Self-assembly provides an information-economical route to the fabrication of objects at virtually all scales. However, there is no known algorithm to program self-assembly in macro-scale, solid, complex 3D objects. Here such an algorithm is described, which is inspired by the molecular assembly of DNA, and based on bricks designed by tetrahedral meshing of arbitrary objects. Assembly rules are encoded by topographic cues imprinted on brick faces while attraction between bricks is provided by embedded magnets. The bricks can then be mixed in a container and agitated, leading to properly assembled objects at high yields and zero errors. The system and its assembly dynamics were characterized by video and audio analysis, enabling the precise time- and space-resolved characterization of its performance and accuracy. Improved designs inspired by our system could lead to successful implementation of self-assembly at the macro-scale, allowing rapid, on-demand fabrication of objects without the need for assembly lines.

Self-assembly and self-organization are among the most peculiar, puzzling aspects of life. They occur at all scales: proteins, viruses, living cells, multicellular organisms, and swarms or societies of multicellular organisms. All these systems are comprised of interacting discrete parts that are attracted to each other in defined ways, leading to the formation of a global structure or pattern.

Compared with these natural systems, human technology still relies almost entirely on assembly lines to build complex things. Assembly lines require immense amounts of information, either in the form of trained personnel or in the form of automatic assembly machines, which in turn are also built on assembly lines, which are by themselves packed with information almost ad infinitum. On the other hand, self-assembly offers an information-economical route to complex fabrication. The successful synthesis and widespread implementation of self-assembly at the macro-scale would arguably revolutionize technology as we know it. However, although programmable self-assembly has been produced in a variety of contexts from the molecular to the micrometer scales^1^,^2^,^3^,^4^,^5^, no known algorithm can program macro-scale objects to self-assembly from their basic bricks.

The challenge of macro-scale self-assembly has been greatly simplified by implementing it in one or two dimensions. A classical demonstration of macro-scale self-assembly used flat bricks sliding on a 1D track^6^. Various works demonstrated assembly in 2D (or 3D approximations of 2D systems), for example on a surface or at the liquid-air interface^7^,^8^. In some cases, the system relied on an external information seed, in the form of templating surfaces^9^.

In contrast to these cases, self-assembling 3D systems must solve the complexity of infinite degrees of freedom. In three dimensions, the parts engage each other from all possible angles, orientations, and velocities, and they must keep testing configurations until the correct one is found. For this, the system needs to be constantly perturbed by external energy, and the configurations need to be carefully designed to allow only the correct one to survive these perturbations. This energy is indispensible for preventing the system from locking in local minima and enabling the real-time correction of assembly errors. Few studies reported self-assembly in 3D, particularly for the fabrication of objects characterized in a high degree of symmetry^10^.

The aim of this study was to demonstrate programmable self-assembly of solid, complex, asymmetric objects in 3D. Our approach was inspired by perhaps the most intriguing example of molecular self-assembly of complex objects in 2D and 3D - DNA origami^11^,^12^,^13^,^14^. In scaffolded DNA origami, the folding of a long ‘scaffold’ DNA strand, typically several thousands of bases long, is guided by several hundreds of short ‘staple’ oligonucleotides into an arbitrary shape based on Watson-Crick base pairing. The scaffold-staples mixture is subjected to a temperature-annealing ramp going from as high as 95 degrees down to room temperature, providing the molecular kinetic energy required for error-free, high-yield assembly. A diverse gallery of nanoscale shapes has been successfully fabricated using scaffolded DNA origami in the past few years^15^,^16^,^17^.

Self-assembly by scaffolded DNA origami is non-algorithmic in the sense that the number of parts in an object is equal to the number of unique part types. In DNA origami each strand is unique, and has a unique position within the assembled object, which is hardwired into its sequence. Designing scaffolded DNA origami shapes has been simplified recently by the introduction of computer-aided design (CAD) tools such as caDNAno^18^. In this example, caDNAno receives as its input a shape outline defined by the user, geometrically subdivides the shape into the discrete staple strands, and computes their sequences based on the scaffold strand sequence provided by the user.

Inspired by scaffolded DNA origami, we designed a system based on the following analogies:

a) Instead of caDNAno, an arbitrary 3D object is subdivided into discrete bricks by Delaunay triangulation.

b) Instead of DNA sequences, the identity and location of each brick in the object is encoded by complementary sets of topographic cues imprinted on each face of each brick.

c) Instead of Watson-Crick hydrogen bonding, attraction between complementary bricks is provided by permanent disc magnets embedded in each face in complementary orientations (N-S, not N-N and not S-S).

d) Instead of a temperature-annealing ramp, the brick mixture is subjected to robust orbital or planar agitation starting as high as 350 rpm and decreasing down to zero.

In this paper we describe an efficient algorithm for meshing an object into bricks that can self-assemble. Moreover we show the detailed, time- and space-resolved assembly process of objects thus designed. Our design could serve as a promising starting point for improved systems with industrial applications.

## Results

### Object and brick design

3D objects can be meshed into discrete 3D parts in infinite ways. We chose to prototype this process by using tetrahedra, which are the simplest space-filling objects. To do this we used Delaunay tessellations, which are a special type of triangulations of a set of points in space^19^. Delaunay tessellations have unique features that make them useful in a wide variety of scientific and engineering applications. In particular, they tend to avoid skinny triangles and can be computed quickly with known fast algorithms. These properties make them ideal for meshing 2D and 3D domains, and indeed they are used extensively in meshing of such domains for engineering purposes such as finite element analysis. Delaunay tessellations of a convex object can be done by sampling points on the boundary of the object and inside it and computing the Delaunay triangulation of the sampled points. For a non-convex object, a more sophisticated algorithm should be used, which uses Delaunay triangulations as a basic procedure. For these cases, a more careful sampling of points is required to maintain the boundary of the object. Furthermore, since the Delaunay triangulation results in a triangulation covering the whole convex hull of the object, excess tetrahedra need to be removed.

**Figure 1:**
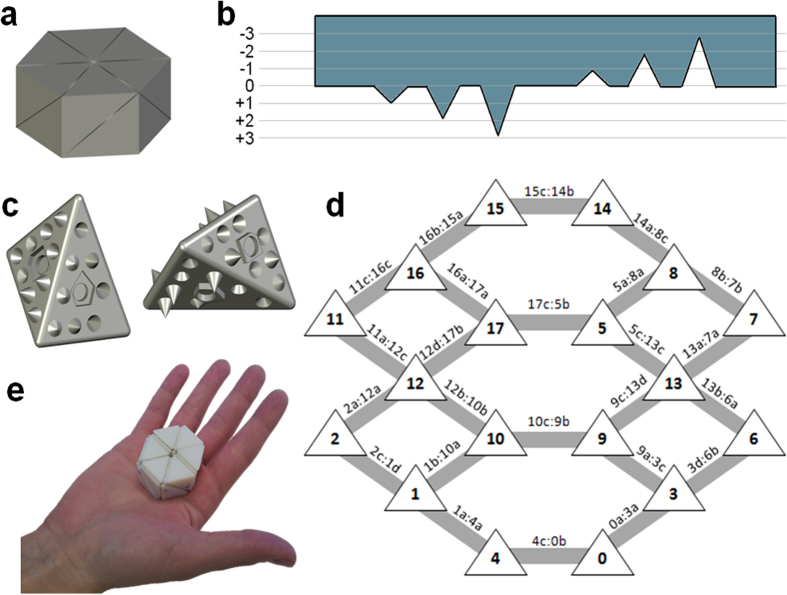
Object and brick design and fabrication. (**a**) The prototype assembly object, a cylinder with radius and height of 15 mm. Delaunay tessellations resulted in a subdivision of the polygon into 18 tetrahedrons from 3 different types. (**b**) Schematic representation of conical cue heights. There are 7 different heights: –3.0, –2.0, –1.0, 0, +1.0, +2.0 and +3.0 mm. (**c**) A tetrahedral brick (number 13) from 2 different views. Each face has a unique topography. Our topography design consists of 10 cues per face. The nine peripheral cues are conical. The central cue can have two different pentagon shapes and two different heights: +1 or −1. In the central cue there is an additional round cue, in which a permanent disc magnet is embedded. (**d**) A graph describing the whole object composed of its building bricks. Each node represents a brick and each edge represents a face-face interaction between 2 bricks. Out of all 18 bricks, there are 2 bricks that interact via all 4 faces, 8 bricks that interact via 3 faces and 8 bricks that interact via only 2 of their faces. (**e**) The final object printed and assembled.

For our tessellation we used CGAL (Computational Geometry Algorithm Library), a public geometric software library that provides C++ implementations of the most widely used geometric data structures and algorithms. In particular, it has an implementation of 3D Delaunay triangulations and an algorithm for 3D mesh generation, which is based on it. In order to tessellate a cylinder for this study, we sampled points evenly on the cylinder boundary and along its axis and computed the 3D triangulation of the sampled points. The geometry of each resulting tetrahedron was then written into a stereolithography file (STL). Arguably, many complex 3D objects can be subdivided this way (Supplementary note 1). A 3D polygon was chosen for this proof of principle study (Fig. 1a), with Delaunay tessellations producing tetrahedral faces with the following ratios between edges: SQRT(2):1:1, SQRT(2):SQRT(2):1, and 1:1:1. Other irregular tetrahedra could also be produced if desired.

Assembly in our system is driven by complementary cue sets imprinted on the face of each part. The diversity of this set determines the possible number of different parts in the system. However, it depends on the number of different cues that can be distinctly and robustly packed on each part, which in turn depends on other factors such as the cue shape and material hardness. Our design consists of 10 cues per face. The nine peripheral cues are conical and are of 7 different heights (−3.0, −2.0, −1.0, 0, +1.0, +2.0, +3.0 mm) (Fig. 1b). The central cue can have two different shapes and two different heights (Fig. 1c). A permanent disc magnet is embedded within this cue. Altogether, the 10 cues on each face (specifically, the faces participating in brick interactions) form a unique topographic signature, and to which there exists a single complementary topography elsewhere in the system. The central cue also ensures correct orientation between the bricks and prevents connections that are not fully oriented face-to-face. Supplementary Note 2 shows the complete topography set used in this system. Our cue layout allows translation of an object into 72 different interactions pairs, as explained in Supplementary Note 3.

To complete the design process, the whole object was modeled as a graph in which the bricks form the nodes while face-face interactions form the arcs (Fig. 1d). The bricks were 3D printed and disc magnets were glued at the center of each brick at the specific magnetic field orientation. Figure 1e shows the fully and correctly assembled object.

### Two-brick assembly

**Figure 2:**
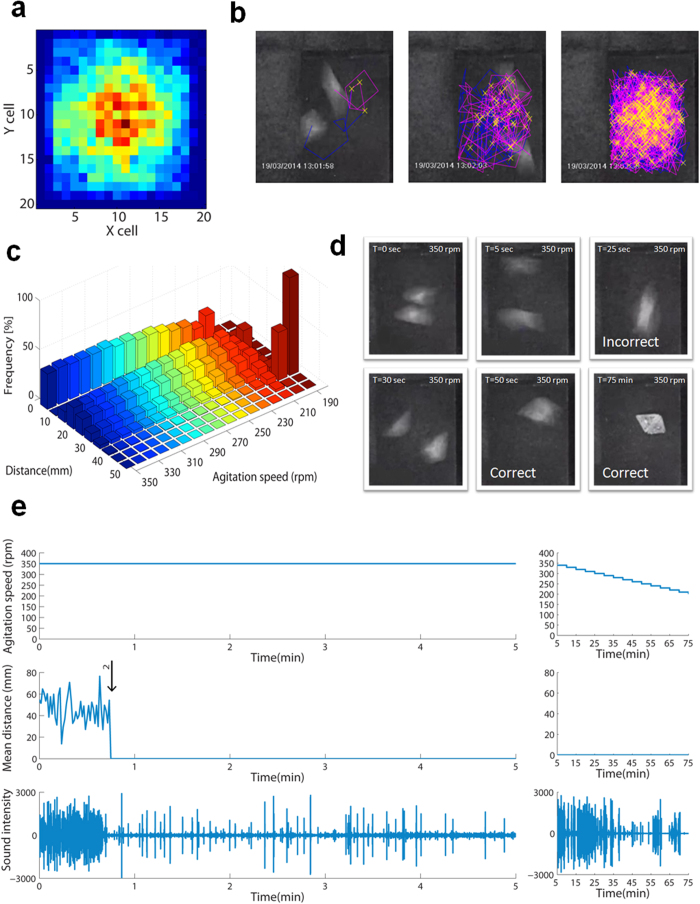
Two-brick assembly. (**a**) Distribution of brick location in the chamber, calculated from observed movement of the brick during a 2-minute agitation at 350 rpm. Color scale represents fraction of time spent in that cell, red = 0.8, blue = 0.0. (**b**) Snapshots from video tracking of 2 bricks (t = 0, 5, 45 sec). A blue or pink line traces each brick’s movements. (**c**) Distribution of distances between bricks vs. agitation speed. Bricks collide (distance 0) approximately 42% of the time. Otherwise the distances show skew normal distribution with an average of ~20 mm, excluding the distributions bellow 210 rpm, in which collisions are rare and distance between bricks is fixed to specific distances. (**d**) Snapshots from the assembly process of 2 bricks with magnets. Incorrect matches break down while correct matches are stable. (**e**) Spatiotemporal tracking of the assembly process of 2 bricks. The top graph describes the agitation speed changes; the middle graph represents the mean distance between the brick centroids. Values were obtained by calculating mean distance for every 30 frames (second). The bottom graph shows amplitude of collision sound audio.

We began exploring the system by placing two bricks without magnets in a closed chamber screwed to an agitating incubator, and monitoring their movements in response to varying agitation speeds going up to 350 rpm. Bricks spent most of the time around the middle, driving higher collision efficiency in that area (Fig. 2a). The same result was obtained using lower velocities as well. Video tracking of the collisions confirmed this result (Fig. 2b). Collision efficiency was confirmed by measuring the distribution of distances between bricks, which showed that the bricks collide approximately 42% of the time. Otherwise the distances show skew normal distribution with an average of ~20 mm (Fig. 2c). Supplementary note 4 details the analysis of displacements, velocity and kinetic energy as a function of agitation speed.

In DNA origami, accurate folding takes place by subjecting the part mixture to a temperature-annealing ramp. Analogous to this, we designed the assembly sequence to begin at the top agitation level (350 rpm), decreasing at a rate of 10 rpm per 5 min. The entire process was monitored simultaneously by video and audio, with collision sound audio clearly indicating correct assembly events (Supplementary note 5). Almost all pairs of bricks assembled at 350 rpm within less than 1 min (Fig. 2d,e. A movie of 2 bricks assembly is available at http://youtu.be/6SqoSARKQPg).

### Global self-assembly

Finally, we tested assembly of the entire object (18 bricks) in our system. Here two issues were critical, the concentration of bricks and brick copy number. In previous experiments we have defined the concentration as the ratio of brick pixel area to total pixel area, using area as an approximation for volume. Concentration was critical for optimal assembly. Two bricks usually required less than 1 min to correctly assemble in a small area (64 cm^2^, concentration of 0.06) and approximately 10 min in a medium area (128 cm^2^), but did not efficiently assembled in a large area (400 cm^2^). However, when concentration was larger than a critical value of ~0.5, the space became too small for efficient mixing.

**Figure 3:**
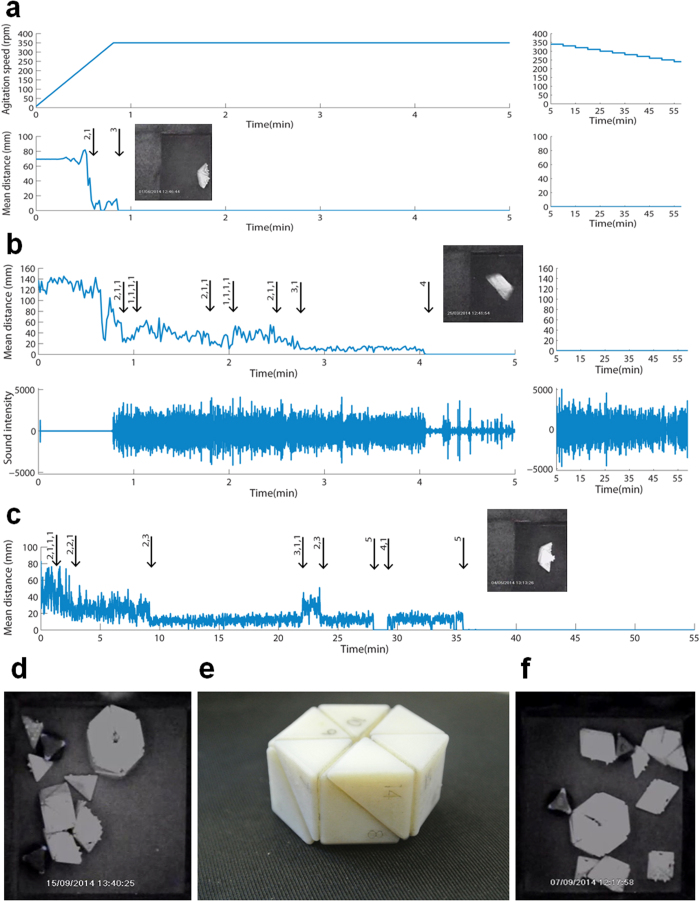
Self assembly of the whole object. (**a-c**) Graphs describing the assembly process of 3–5 bricks, in terms of agitation speed, mean distance between brick centroids, and audio amplitude profile of the process. Arrows indicate assembly events (attachment or detachment), numbers denote complex components. (**d**) 2 sets of the whole object (36 bricks) were inserted in the chamber and after 2.5 hours in a constant speed of 320 rpm one set was assembled perfectly (**e**). (**f**) 2 sets of the whole object lacking one copy of brick 12 (35 bricks) were inserted in the chamber and after one hour one set assembled perfectly, except for one missing brick, brick 12.

We proceeded from 2 bricks to 3, 4, 5 and on, in a small area. Assemblies took a few minutes but gradually required more time to complete as object complexity increased (Fig. 3a–c. A movie of 3 bricks assembly is available at http://youtu.be/jr7oXPkqZsI. A movie of 4 bricks assembly is available at http://youtu.be/XZj_Se2ML_Y. A movie of 5 bricks assembly is available at http://youtu.be/3-POX7TM7w4). The global assembly of all 18 bricks was performed with 2 copies of each brick in a larger area to avoid reaching the critical concentration value. Strikingly, the assembly completed within up to 2.5 hours at a yield of 50% reproducibly (Fig. 3d–e. A movie of global assembly is available at http://youtu.be/nuoY3NWWEPA). The system was robust enough to allow assembly in the absence of one brick (Fig. 3f. A movie of global assembly missing one brick is available at http://youtu.be/kmSOO_3m9yk). Our findings suggest that it is possible to assemble this way various objects from within a large set of bricks, where each object is formed by a subset.

## Discussion

Self-assembly of complex objects at the macro-scale is an important scientific and technological challenge. In this work we present a design approach that draws analogies to the self-assembly of DNA strands in DNA origami^11^, and an assembly system that makes use of camera and microphone for spatiotemporal monitoring of the process. Our goals in this study were mainly two: first, to propose an environment for prototyping and optimizing schemes for self-assembly in the macro-scale; and second, to describe a particular design that makes use of topographic cues for directing the assembly of an object.

The use of complementary topographies has two main advantages. First, topography is scalable, providing large diversity of bricks by making use of various shapes and heights. However, increasing diversity requires the cues to be packed with higher density on the faces of each brick, hence they must be smaller. For this to be feasible, the cues must be made of hard materials that are compatible with the agitation protocol optimized for the assembly of a particular object. Still, modern manufacturing technologies enable this feat, and allow most types of parts to be imprinted with such cues.

Second, topography mechanically strengthens the binding between bricks. Once the bricks are correctly assembled, complementarity between cues prevents the bricks from sliding relative to each other and drastically limits motion in other directions. Thus, topography provides an additional component of binding strength rather than just specificity.

The choice of attractive force depends mostly on object scale and functionality. Molecular recognition is sufficient to drive self-assembly at the nano- and micro-scales as previously demonstrated. Other forces might be required at the macro-scale. A good example is magnetic forces, which were used in this study, providing both specificity (via magnetic pole orientations) and strength. However, permanent magnetic fields are incompatible with objects and functions such as memory devices in computers, which therefore might require other methods such as capillary forces or adhesive surfaces. Objects that are expected to withstand high shear, bending or torque moments, such as working tools (hammers, screwdrivers, etc.), might require a combination of forces to be efficient. We are currently investigating the intriguing possibility of using electromagnetically triggered cyanoacrylate applied on each face in the system. Thus, topography and magnetic fields provide the first assembly step, which is followed by activation of an adhesive that permanently fixes the object in its final state.

We observed inherent differences in binding efficiencies between brick pairs (Supplementary note 6). This apparent drawback provided an intriguing insight and potential solution to a central challenge of complex 3D self-assembly. In contrast to hollow 3D objects, such as the viral capsid model designed by Olson and colleagues^10^, the self-assembly of solid objects needs to solve the problem of assembly sequence: how to make sure the internal parts assemble before the external ones. Our experiments showed that differences in binding efficiencies could be utilized to drive assembly sequences if they are organized in a gradient, with the internal parts the most efficient. Once these assemble correctly, they serve as seed for the assembly of the external parts.

Interestingly, our experiments revealed striking similarities between self-assembly at the macro-scale and nano-scale, with scaffolded DNA origami exemplifying the latter. The agitation protocol was designed in an analogy to the thermal annealing ramp used for DNA origami assembly. This protocol typically starts at an arbitrarily high temperature such as 95 °C or 80 °C, and decreases to room temperature in a sequence which, in some cases, takes days to complete^20^. However, a recent study demonstrated that most of the assembly process occurs over a very short period of time at a certain critical temperature that depends on the assembled object^21^. Similarly, our agitation protocols were designed to start from high speed (350 rpm) and decrease to zero over a long period of time. However, our spatiotemporal tracking showed that the entire assembly occurred at a particular speed and often took seconds to complete. This equivalency bolsters the hypothesis that self-assembly is a critical phenomenon similar to phase transition.

Another interesting similarity was the dependence on concentration. Efficient DNA origami assembly depends on strand concentration in the folding reaction. In our system, concentration was defined as ratio of bricks to area (since volume is not a significant dimension in the setup). When the area was too large, brick assembly was inefficient and often did not take place at all. This could be solved by introducing “solvent” bricks – inert, magnet-free bricks that do not participate in the assembly itself, but contribute to assembly by colliding and fixing incorrect assemblies (Supplementary note 7). This concept can be extended as a general principle in future systems.

Brick copy number was hypothesized to play a role as well. In DNA origami, as well as in other forms of molecular self-assembly, many copies of each brick exist, resulting in many copies of the assembled shape at a certain yield which depends on the shape but can be as low as 5%^11^. Our global experiments demonstrated rapid (1 hour) assembly at 50% yield using 2 copies of each brick. It is interesting to note that as a manufacturing strategy, even low yield self-assembly could be efficient, having eliminated the costs associated with assembly lines. Moreover, after the correctly assembled objects are removed, the remaining bricks can be passed through a new agitation cycle without further procedures.

Arguably, ideal assembly would occur with the system equally agitated at all directions. However, in our system, as in any other macro-scale system, gravity imposes a constant bias. Efforts are underway to investigate the behavior of our system in zero gravity conditions. However, it should be noted that any realistic assembly process as a technology would include gravity, and it should thus be accounted for.

In conclusion, we describe here a system for the design and investigation of self-assembly of complex, macro-scale 3D objects, an important challenge scientifically and technologically. Our approach could be adopted for designing improved systems, and produce data and insights that could contribute to the study of self-assembly as a physical phenomenon which is still not well understood.

## Materials and Methods

### CAD and computational geometry

3D objects were subdivided to tetrahedral bricks by Delaunay triangulation^19^ using CGAL^22^. Topographic cue design was done on the bricks using DS SolidWorks 2012 running on a Hewlett-Packard PC. Bricks were printed on a Stratasys/Object Eden 250 3D printer using either VeroWhite or DurusWhite as printing materials. Support resin was removed from the finished models by water jet followed by soaking in 5% NaOH for 2 hours at room temperature and finally a thorough wash with water. Neodymium-Iron disc magnets (N45, 2.8 mm × 0.8 mm) were glued to the bricks using commercial cyanoacrylate glue.

### Topography design

Brick topography was programmed to give each pair of matched faces complementary cue sets (profiles). In order to maximize the positive free energy gap between a perfect match and imperfect matches, while enabling a construction of a large group of unique profiles, we determined the threshold of identity between profiles to be up to 66% (the algorithm relates identity to “real” matching, e.g. +2 mm cue facing a −2 mm cue, as well to “fake” matching as +2 mm cue facing a −3 cue). Another constraint used in the algorithm is on the number of flat topography (height 0) permitted on each profile. Flat topography adds to the diversity but cuts back from the mechanical binding strength between bricks. The threshold we used is up to 3 flat cues in a profile.

### Assembly system setup

The setup included a custom-built box (Supplementary Note 8). The box was screwed to the stage of a Multitron orbital shaker with speed control ranging up to 400 rpm. Agitation speed was raised or lowered in real time with 1 rpm increments. Video images were recorded using tiny color camera (1 gram 520 TVL, single chip 1/3 CMOS, sensitivity of 0.008Lux/F1.2, rate of 30 fps). Audio was recorded by a capacitor microphone with a pre-amplifier with adjustable intensity. Both camera and microphone were connected to a wireless transmitter (Sanyo Semicon Device), and data were stored on a remote Provision H.264 Digital Video Recorder.

### Data analysis

Video data was analyzed in MATLAB using a custom-written code as explained in Supplementary Note 9.

Audio data was analyzed using WavePad. Discrimination of collisions from shaker’s rotor noise was carried out by a High-Pass Filter (2700 Hz) and equalizer (10% for <2500, 100% for >=2500). A noise reduction procedure preceded and followed each operation.

Finite element method was used for the magnetic simulations. Interaction simulations were done in Cobham Opera3d utilizing static simulations. The analysis of the data was done in MATLAB.

### Statistical analysis

Each experiment was repeated at least 10 times. Assembly yield was calculated as the percentage of successful assemblies out of total experiments.

## Additional Information

**How to cite this article**: Hacohen, A. *et al*. Meshing complex macro-scale objects into self-assembling bricks. *Sci. Rep*. **5**, 12257; doi: 10.1038/srep12257 (2015).

## Supplementary Material

Supplementary Information
