# Supplementary material for: Meshing complex macro-scale objects into self-assembling bricks
Source: Sci Rep. 2015 Jul 30;5:12257. doi: 10.1038/srep12257 (PMC4520187; doi:10.1038/srep12257)
Supplement: Supplementary Information [file srep12257-s1.docx]

**Meshing complex macro-scale objects into self-assembling bricks**

**Adar Hacohen^a^, Iddo Hanniel^b^, Yasha Nikulshin^c^, Shuki Wolfus^c^, Almogit Abu-Horowitz^a^, Ido Bachelet^a,d^**

a) Faculty of Life Sciences and the Institute of Nanotechnology & Advanced Materials, Bar-Ilan University, Ramat Gan, Israel.

b) Faculty of Mechanical Engineering, Technion Israel Institute of Technology, Haifa, Israel.

c) Department of Physics, Faculty of Exact Sciences, Bar-Ilan University, Ramat Gan, Israel.

d) Address for correspondence: Ido Bachelet, Room B337, Building 206, Bar-Ilan University, 1 Anna & Max Webb St., Ramat Gan 52900, Israel. Phone: +972 3738 4312. Email: ido.bachelet@biu.ac.il

adar.hacohen@gmail.com

iddohanniel@gmail.com

yasha.nick@gmail.com

Shuki.Wolfus@biu.ac.il

almogit@gmail.com

**Supplementary note 1**

Subdividing 3D objects into discrete building blocks is a well-studied problem with many applications in science and engineering. For example, finite element analysis begins with such a subdivision and performs the analysis on the tessellated object. Thus, there is a vast body of knowledge on implementing such tessellations for complex real-world geometric shapes.

The main subdivision types appearing in the literature are tessellations by hexahedra and by tetrahedra (triangles and quads in 2D). The former have the advantage of a simpler representation, whereas the latter produce better approximations of the object geometry. In our current work, we used a Delaunay-based tetrahedral subdivision, for which public implementations exist. However, other subdivisions from the literature can also be considered.

It should be noted that these tessellations are approximations in the sense that they generally consist of planar faces, whereas the original shape may have curved boundaries. For many applications and many shapes, such an approximation is sufficient. However, if a smoother representation is required, one can think of ways to adjust our method accordingly. For example, a post-process smoothing of the shape can be applied, similar to processes that are applied to non-smooth 3D-printed objects. Alternatively, when constructing the bricks, the outer faces of the boundary tetrahedra may be constructed with a curved geometry that conforms to the original geometry.


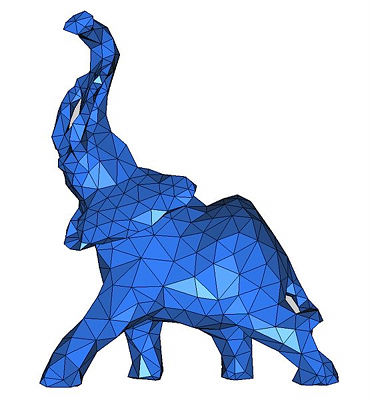


B.


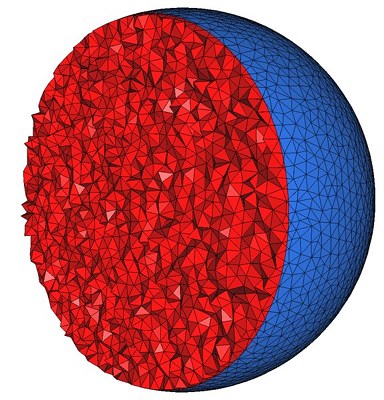


A.

[Type a quote from the document or the summary of an interesting point. You can position the text box anywhere in the document. Use the Text Box Tools tab to change the formatting of the pull quote text box.]

**Supplementary Fig. S1** (A( An example of a cut of a Delaunay triangulation of a sphere. (B) An example of a 3D mesh of a non-convex object (taken from the CGAL documentation).

**Supplementary Note 2**

The complete set of topographic cues used in this system is described in **Table S1.**

table. Colors represent cue heights: Red for+3, light red for -1, green for +2, light green for -2, blue for +1, light blue for -1 and white for 0. The magnets in the central cue are colored in white or in black. Their colors represent direction of the magnetic field.


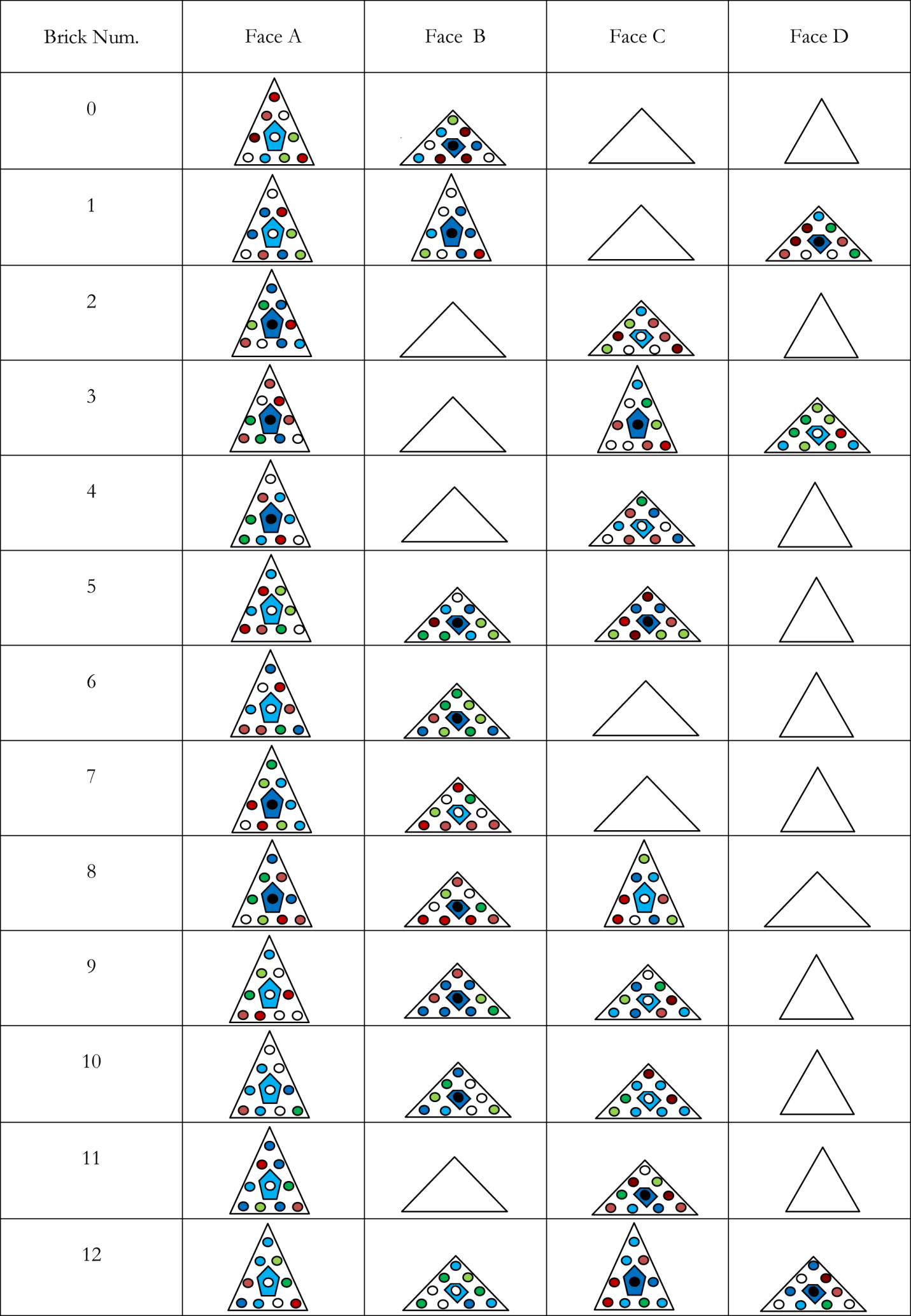
**Supplementary Table S1.**


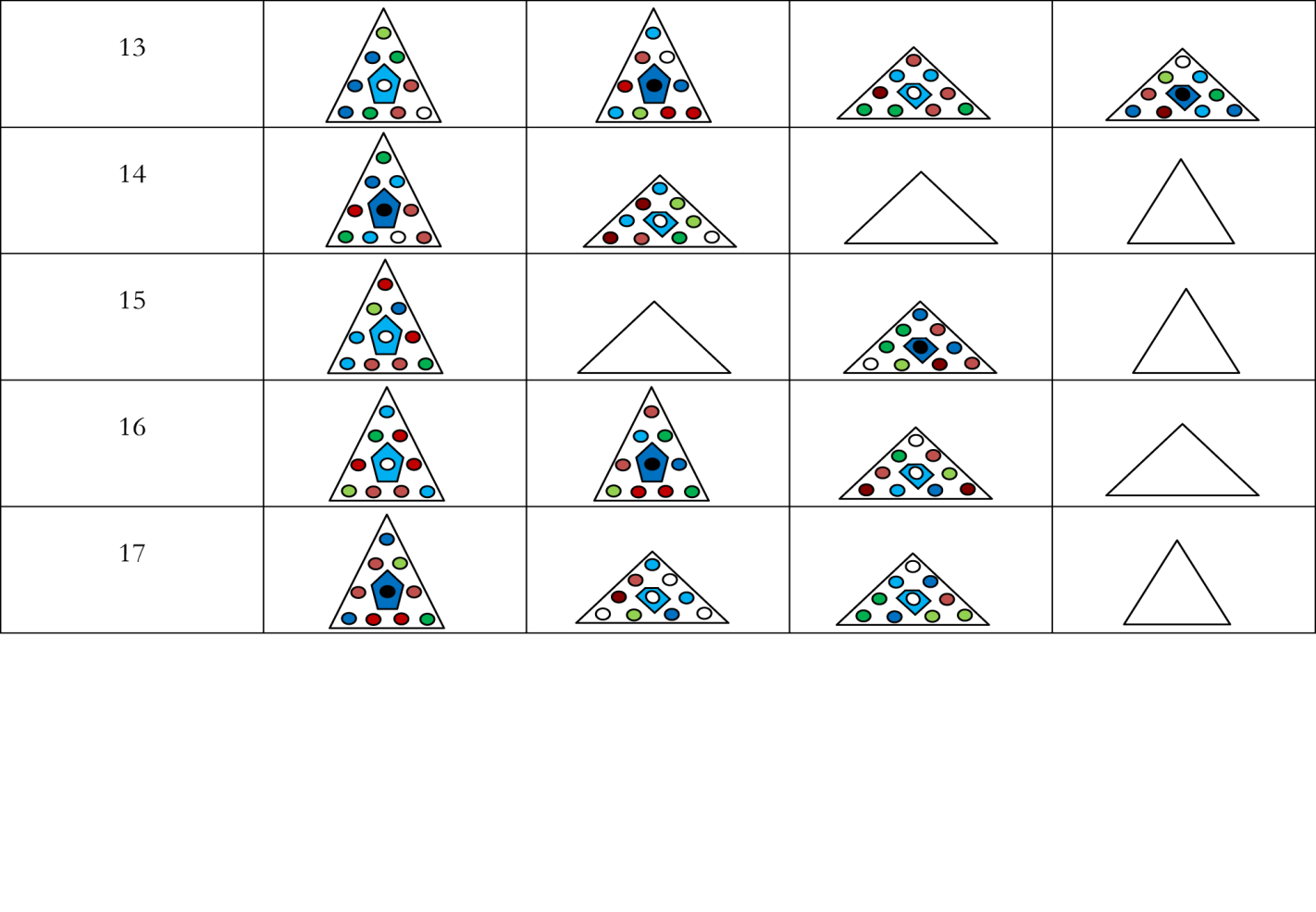


**Supplementary Note 3**

The Interactions in our system can be divided into 3 groups (**Fig. S2**).

First (colored in yellow), are the complementary interactions that represent “Real” matching, i.e. 0/0, +1/-1, +2/-2 and +3/-3. Second (colored in blue), are the interactions that represent “Fake” matching, e.g. -2/+1, -3/-3 and 0/-2. These interactions are not complementary to each other, but their interactions don’t cause a topography gap above the surface of the face and don’t disturb the attachment of 2 faces, and therefore they are considered as “Fake” matching. Third (colored in red), are the non-complementary interactions that create a topography gap above the surface.


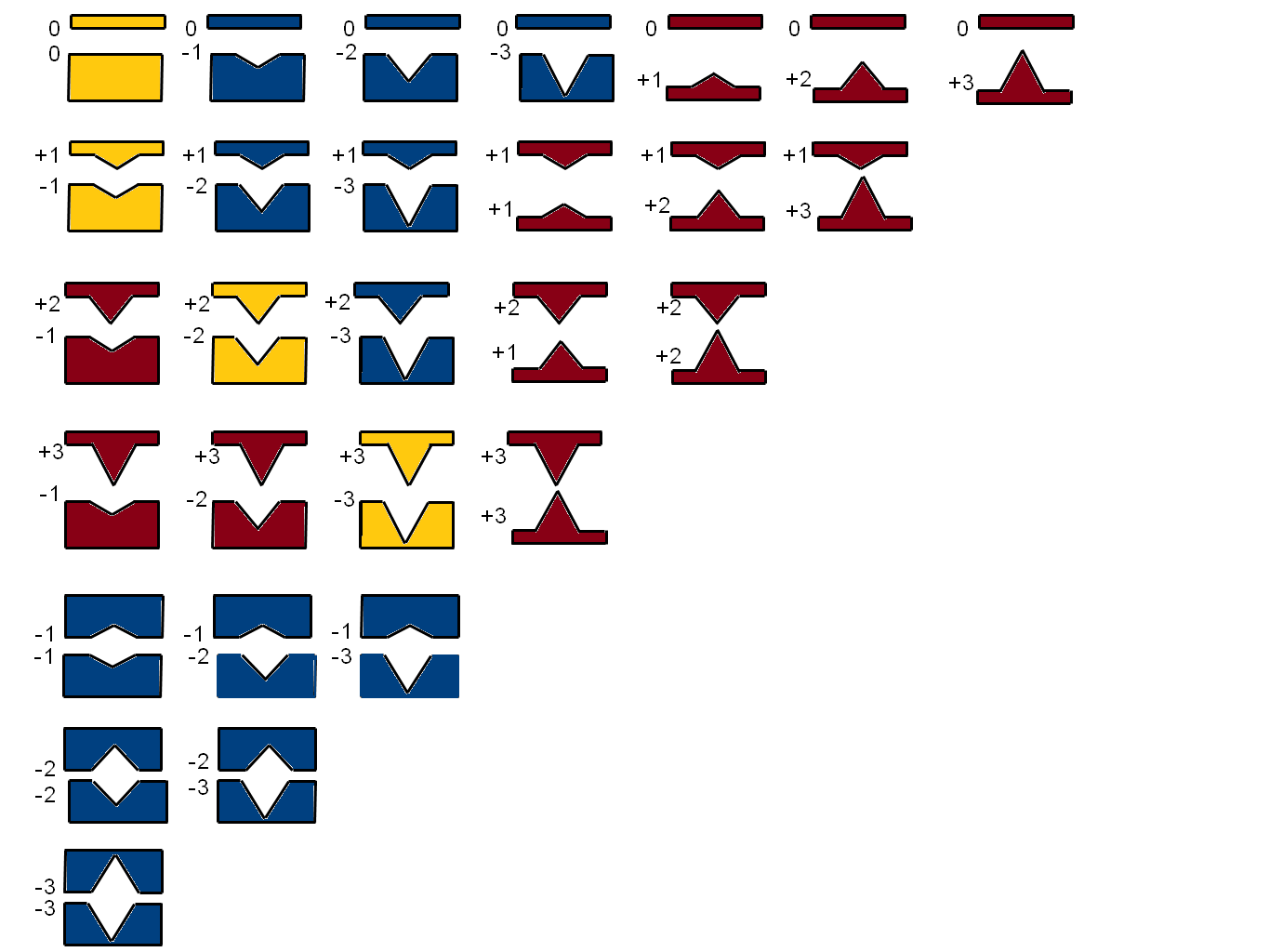


**Supplementary Fig. S2** Types of interactions: Real (in yellow), Fake (in blue) and non-complementary (in red).

Brick topography was programmed to give each pair of matched faces complementary cue sets. In order to maximize the positive free energy gap between a perfect match and imperfect matches, while enabling a construction of a large group of unique profiles, we determined the threshold of identity between profiles to be up to 66% .the algorithm relates identity to “real” matching, as well to “fake” matching. Another constraint used in the algorithm is on the number of flat topography (height 0) permitted on each profile. Flat topography adds to the diversity but cuts back from the mechanical binding strength between bricks. The threshold we used is up to 3 flat cues in a profile.

Taking all these constraints in consideration, our algorithm gave 72 pairs (complementary to each other) of unique permitted profiles. Because our design doesn’t enable matching between 2 faces from 2 different types (different outline triangles), each “type” of face has 72 profiles to use. For example our system has 3 types of faces described by the following ratios: SQRT(2):1:1, SQRT(2):SQRT(2):1 and 1:1:1. The first 2 triangles participate in the interactions while the third doesn’t. All together our system is built from 24 interactions. 12 interactions are between the first triangle type, and the rest 12 interactions are between the second triangle type. We utilized only 1/6 of the permitted profiles. Therefore, theoretically we could create a complex of 6 of our cylinders packed on each other. This way we will use all 72 interactions allowed for the 2 first faces type. The connections between the cylinders are through the third type of face that wasn’t used for interaction in the cylinder. In this packed model we would use only 30 interactions of the third type (6 for each level). All together, 108 bricks with 174 interactions.

In Delaunay triangulation we can control the shapes of the tetrahedrons we receive by the points we sample. So, this gives us quite a big library of profiles that is sufficient to build large objects (as discussed in detail in supplementary note 3).

There are several ways to increase the permitted number of profiles for each face type. First, we can change the coding in a way they we will reduce the “fake” interactions by using different shapes of cues instead of one shape in different heights. Interactions from the type -/- (indentation facing an indentation) or -/0 (indentation facing a flat cue) will remain, but all interactions from the type +/- (protrusion facing an indentation) will be changed to “non-complementary” interactions, what will increase the number of profiles. A second way to increase the number of profiles is by increasing the number of cues on each face. The number of cues on each face is limited by the size of the bricks.

**Supplementary Note 4**

We tracked a video of a single brick in different agitation speeds, starting from 190 and ending 350, with 10 rpm increments. One minute at each speed. In orange, total distance one brick passes as a function of agitation speed. In green, mean velocity of the brick as a function of agitation speeds. In black, kinetic energy of the brick as a function of agitation speed. The energy was determined by the formula E = ½ mv^2.


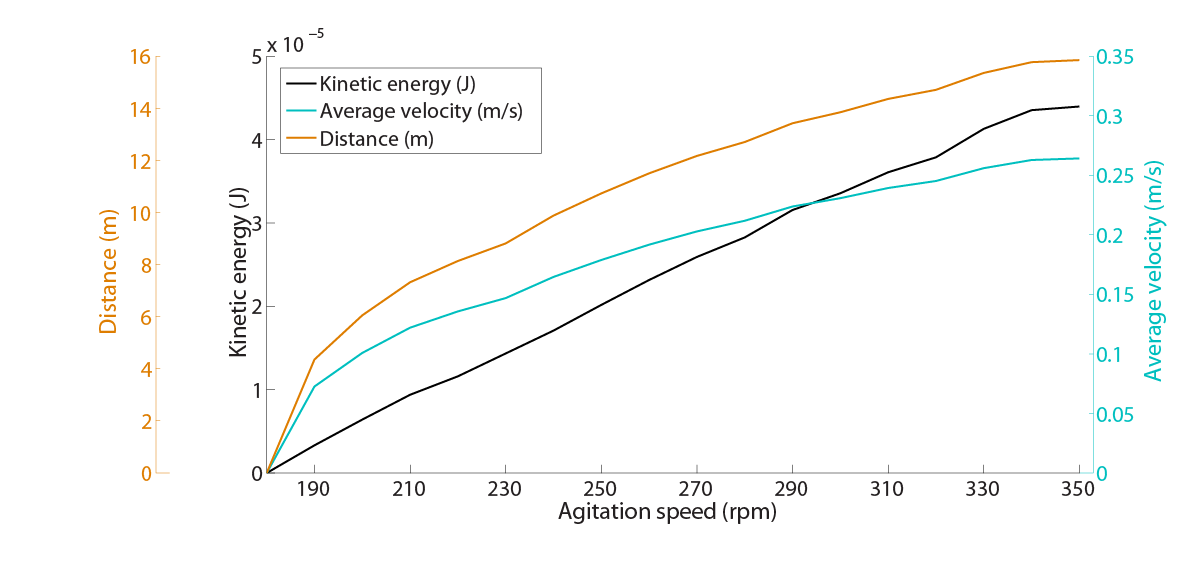


**Supplementary Fig. S3** analysis of displacements, velocity and kinetic energy of a function of agitation speed.

**Supplementary Note 5**

We can notice that as the assembly process progresses the amount of collisions between the bricks decreases, because there are less “free” bricks that can hit other bricks or the boundaries. If a correct assembly occurred the decrease in the collisions will be maintained until the end of the process, and if an incorrect assembly occurred, collision frequency will increase back during the process. The amplitude of the collision sound audio represents the number of collisions and their magnitude. In high rpm collision magnitude will be higher than in low rpm, because of their increased kinetic energy **(Supplementary Note 4)**. We normalized audio amplitude to the assembly process of 2 bricks (see fig 2E of the main text) into number of collisions (**Fig. S4B**).

A.


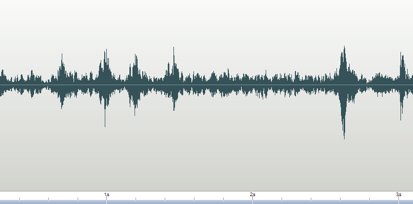


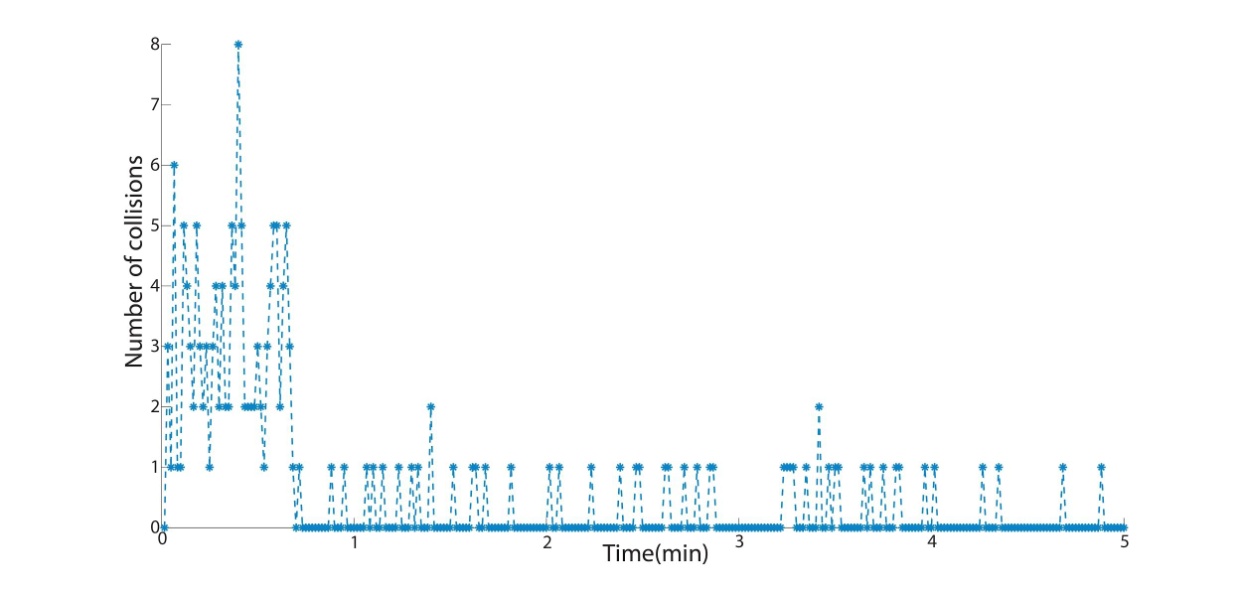


B.

**Supplementary Fig. S4** (A) Record of an assembly process. The collisions can be observed clearly and are pointed by arrows. (B) Graph representing the number of collisions that had occurred between the 2 bricks and between the bricks and the boundaries, in the first five minutes of the assembly process.


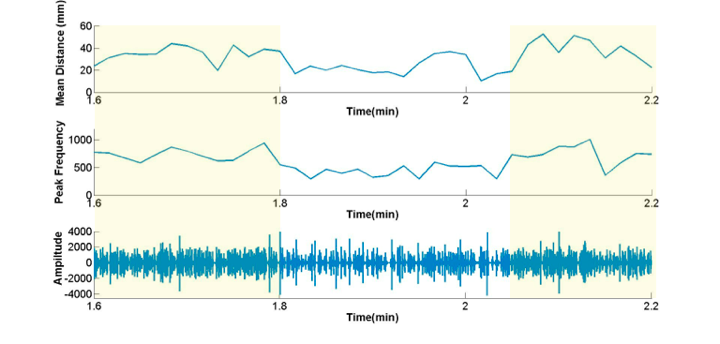


**Supplementary Fig. S5** A graph showing 36 seconds, out of the assembly process of 4 bricks. This fragment represents an event of two single bricks creating a pair complex and breaking up again to single bricks. Yellow regions represent single brick phases. Top graph represents mean distance between brick centeroids, bottom graph shows raw collision sound amplitudes, and middle graph shows calculated peak frequency per second from bottom graph data. Interestingly, strong collisions precede any major conformational change, explaining for example the effectiveness of solvent bricks in driving correct assemblies.

**Supplementary Note 6**

**Interaction simulations and studies.** Finite element 3D models were created and simulated to resolve the interaction between isolated brick pairs. To obtain the single pair interaction the magnetic force between the bricks was calculated using the special derivative of the magnetic pair energy. Bricks were positioned facing each other at a fixed distance of 6 mm between the faces. Then, one brick was kept fixed in space and the other was rotated with the radius vector forming half-sphere in space (6 mm is the minimal distance allowing such relative rotation in our bricks). **Fig. S6** shows the angle dependence of the magnetic force between the bricks, with some pairs showing stronger interaction than others even when aligned face-to-face at the same distance. This is explained by the different sums of projection of magnetic force vectors **(Fig. S6**, **green arrows**) along the radius vector connecting the bricks. This identifies an important design consideration when subdividing objects for self-assembly: the sum of magnetic force vectors should be optimized in all interactions throughout the object, with minimum perturbations.


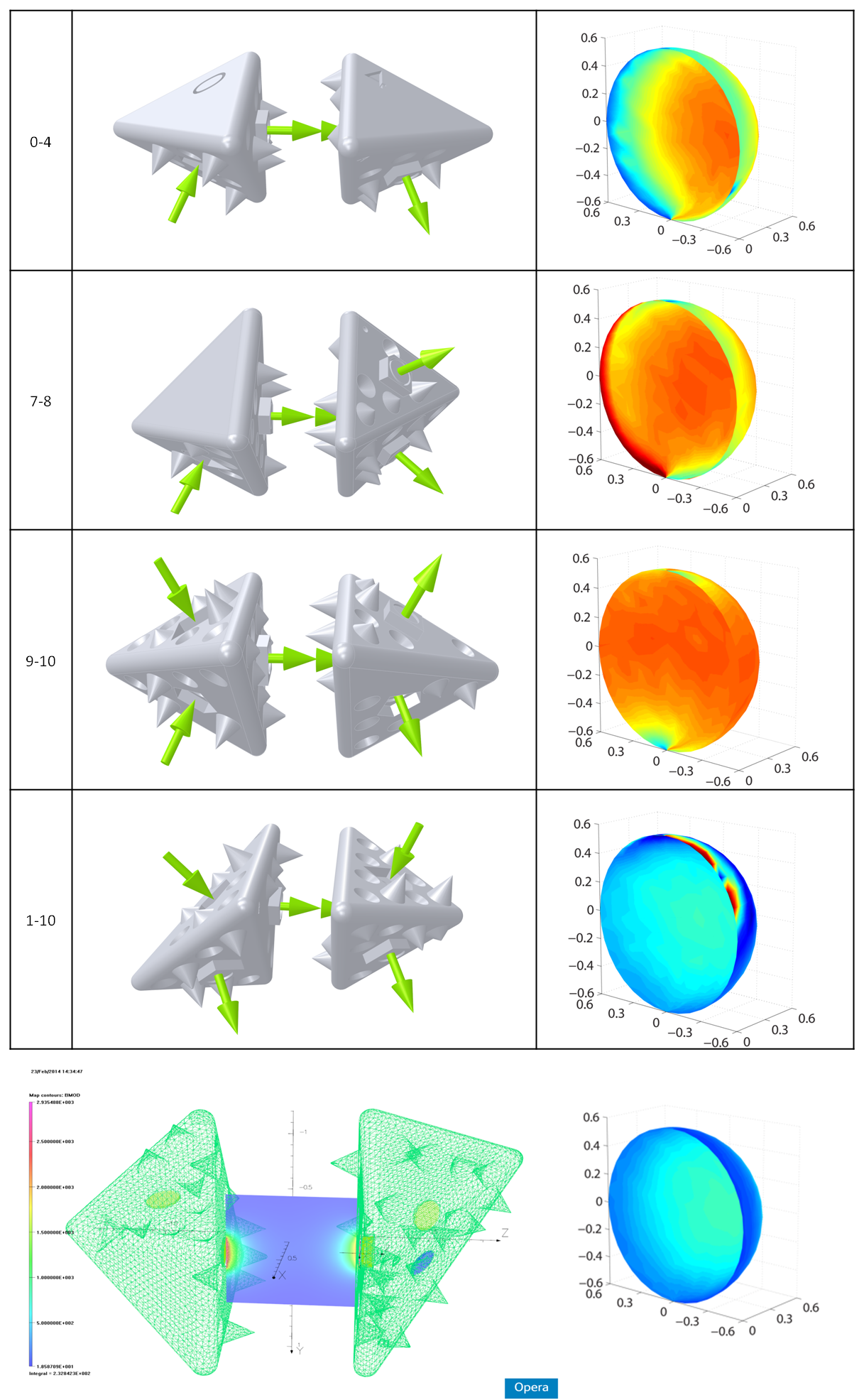


B.

A.

**Supplementary Fig. S6** Magnetic field simulations. (A) Simulation results of magnetic fields between brick pairs. Left column shows the bricks as positioned initially. Right column shows the angle dependence of the magnetic force between the bricks in color-coding (red=strongest, blue=weakest). Green arrows represent the direction of magnetic field dipole. The angle dependence correlates with the sum of magnetic field vectors along the radius vector connecting the bricks. Below the right column is a negative control, derived from a pair of bricks with a magnet on one face only. (B) A Scheme describing our simulations. The faces are parallel to each other in a distance of 1 cm.

**Supplementary Note 7**

As means to improve the assembly process, we introduced “solvent” bricks, analogous to the solvent molecules in molecular self-assembly. These bricks don’t have magnets and are not cued in any way, and their role is to increase the rate of collisions between the functional bricks, enabling them to screen more possible assembly configurations. To test the effect of solvent bricks on suboptimal systems, we added an increasing number of solvent bricks to a system agitating at 250 rpm, in which incorrect assemblies dominated. Addition of these bricks led to two important improvements: it increased the number of collisions by approximately 20%, and it facilitated the breakup of incorrect assemblies, allowing correct ones to occur (**Fig. S7**). The global improvement was significant, although locally some opposite effects were observed. Interestingly, audio analysis clearly showed that a strong collision precedes any major conformational change, visualizing the effectiveness of solvent bricks in driving correct assemblies. The number of solvent bricks can be increased however to a certain optimum of bricks concentration.

B.

A.


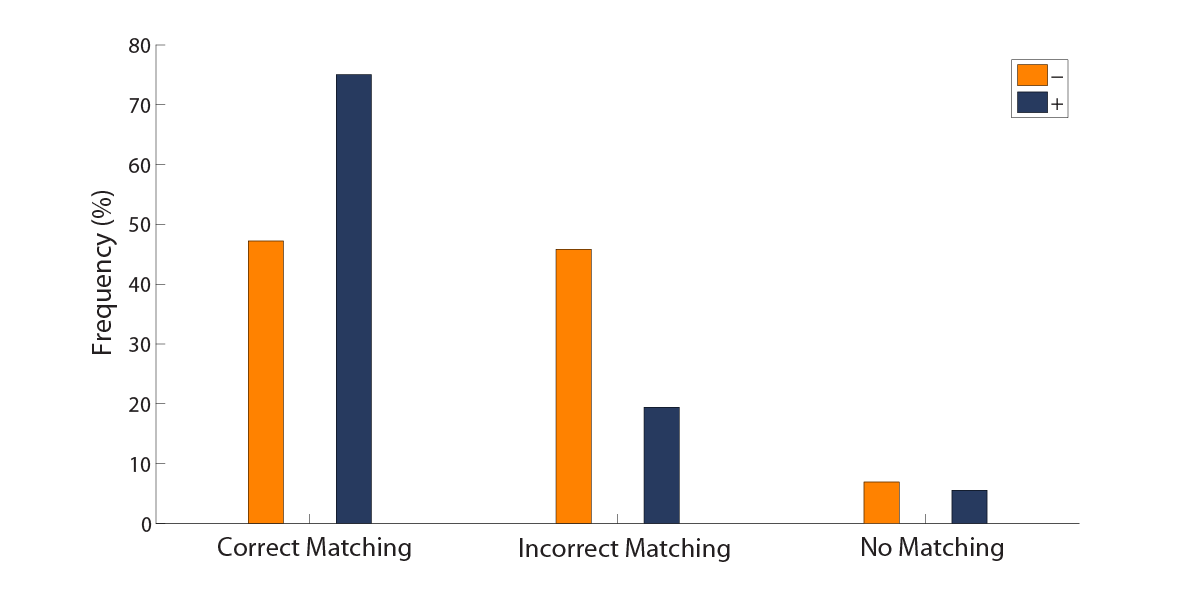


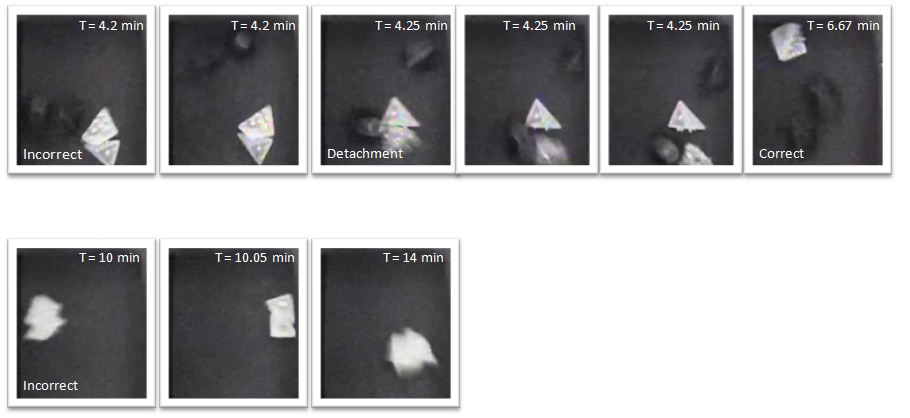


**
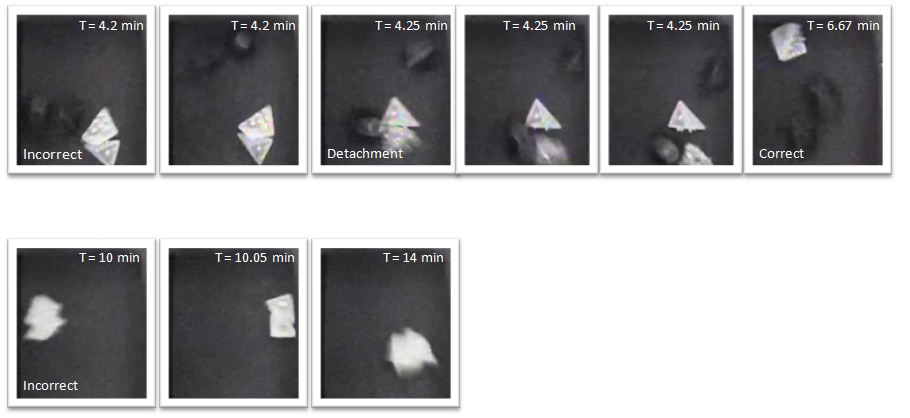
**

C.

**Supplementary Fig. S7** (A) Distribution of brick assemblies in 250 rpm. “–“ indicates experiments with no solvent bricks. “+” indicates experiments with 2 solvent bricks. (B) Screenshots from assembly without solvent bricks. Incorrect assemblies stay stable. (C) Screenshots from assembly that includes solvent bricks. It is clearly shown that the solvent bricks actively break up incorrect assemblies to drive improved global assembly.

**Supplementary note 8**


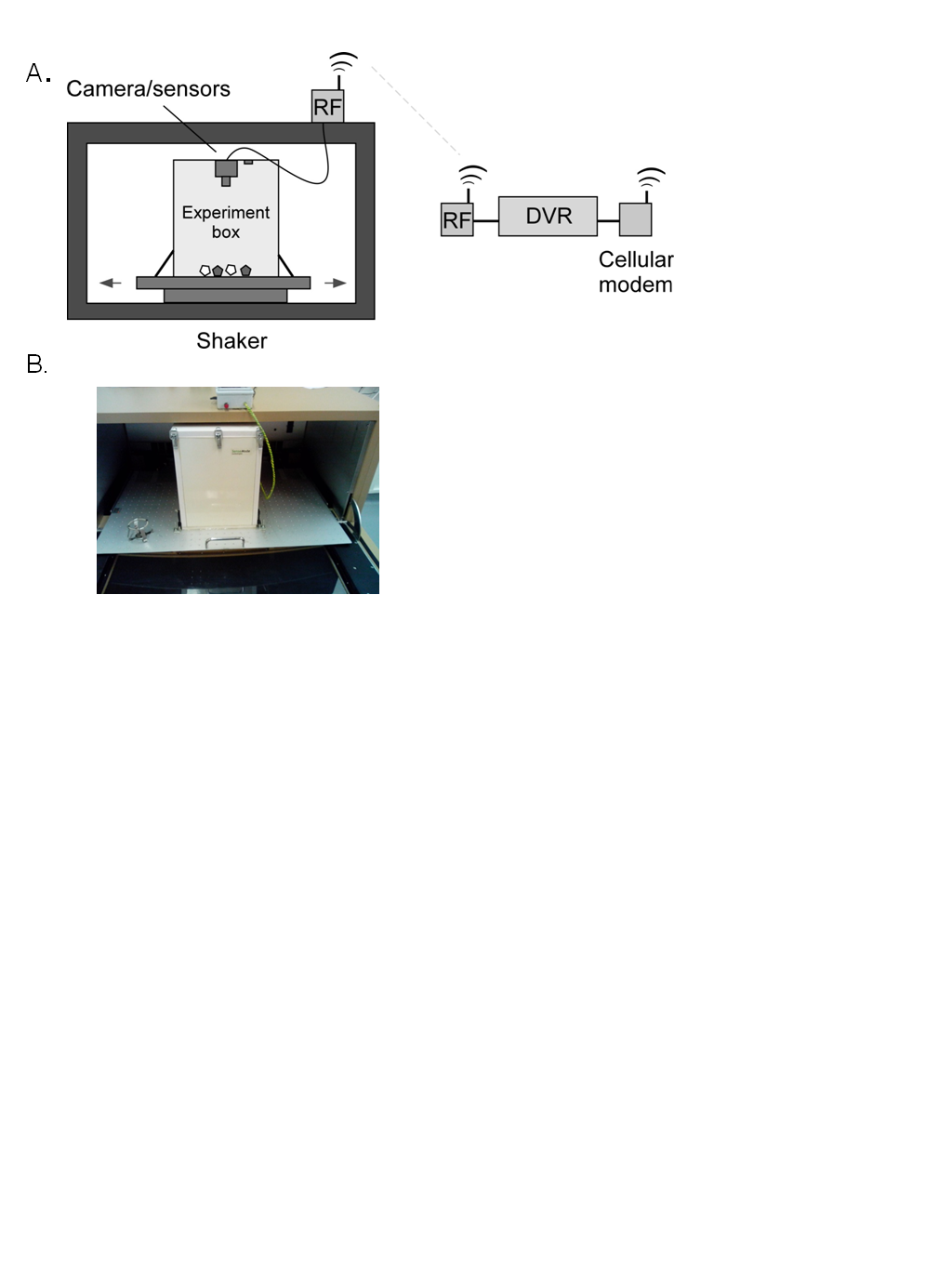


**Supplementary Fig. S8** (A) Scheme of our Assembly system setup. (B) Picture of the Multitron orbital shaker with the assembly box screwed to its stage .The box is built from wood and an acoustic insulation material. Led bulbs were installed On the top of the box, with the ability of controlling their intensity by a regulator. Box dimensions: 27cm (width)*27 cm (depth)*34 cm (height). Wall thickness: 1.5 cm.

**Supplementary Note 9**

Video data was analyzed in MATLAB using a custom-written code. The algorithm of brick tracking can be divided into two parts:

a) Detecting bricks centroids in each frame, including a discernment of how many bricks are assembled in each complex.

b) Associating the detections corresponding to the same object over time.

Brick centroid identification is based on their color (white) and their contrast from the background (black). Finally, blob analysis detects groups of connected pixels. Distinguishing between a single brick and increasing numbers of bricks in a complex is based on the pixels area (**Fig. S9A**).

Associating the detections corresponding to the same object over time, during incidents of attachment and detachment between the bricks, is based on minimum motion. The algorithm determines the matching between each brick to each track that has the highest likelihood to occur. The optimal matching is the matching that gives minimum distances between the detections and their tracks, taking in consideration the probability of an incident (attachment or detachment) to occur or to continue (**Fig. S9B**).


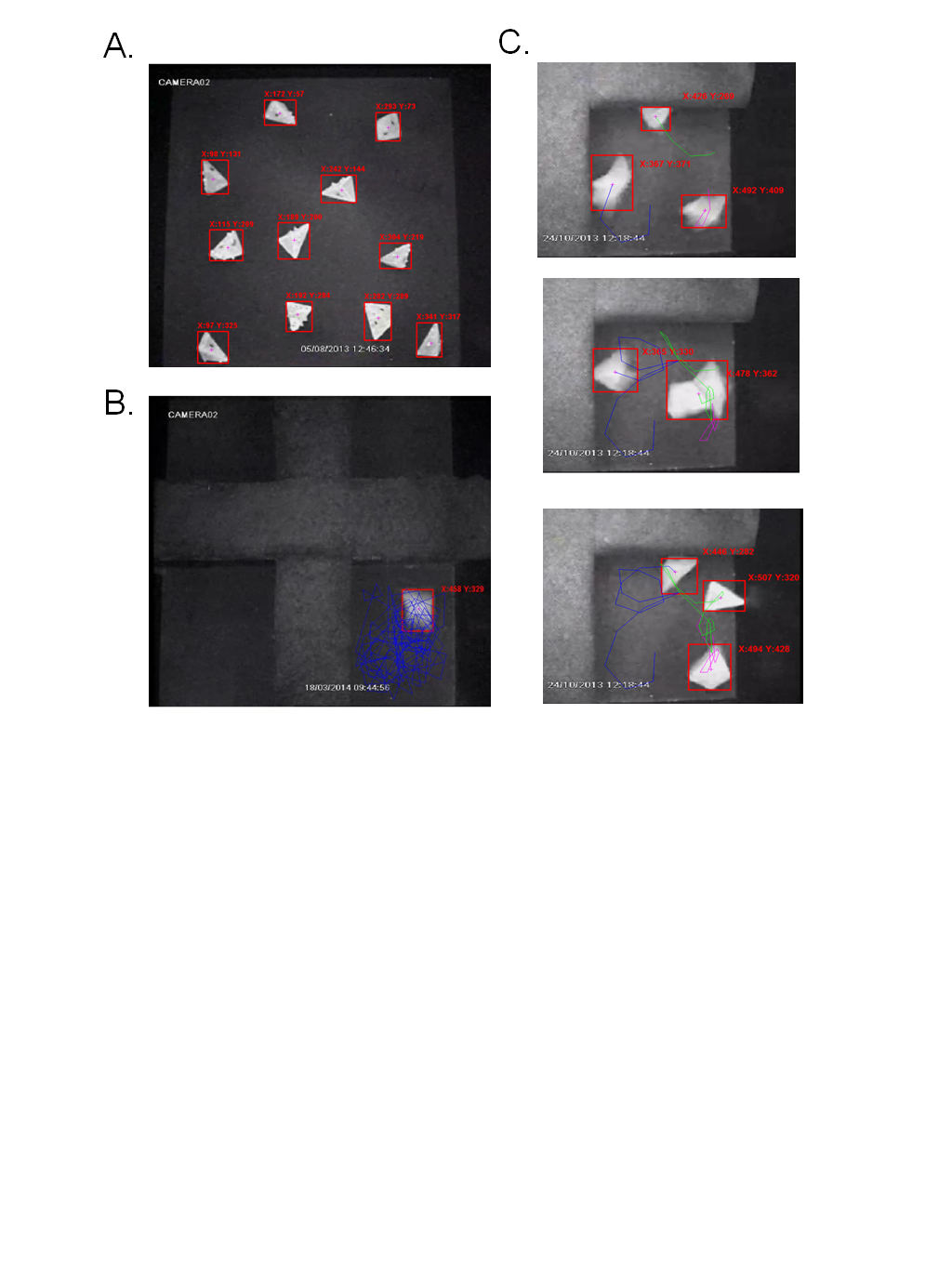


**Supplementary Fig. S9** Brick detection and tracking. (A) Detections of few bricks scattered in the chamber. The coordinates refer to the tetrahedrons centroids which are marked with a ‘+’ sign. (B) Single brick track in blue. The last position of the brick is marked in red. (C) 3 bricks track. Each brick has a specific color that draws his track. In the left snapshot the bricks started to move, without interacting with each other. In the middle snapshot 2 bricks had collided, therefore have the same coordinates. In the right snapshot, the algorithm continued to associate the right track to each brick, along with their separation back to single bricks.

**Supplementary Note 10**

The Average time for assembly grows logarithmically as the number of bricks increase.

In this graph we can see our results up to 18 bricks (the whole shape). By extrapolation we can predict the time it will take for a system comprised of larger numbers of bricks. For example 100 bricks are estimated to require about 4 hours and 20 minutes. And 1000 bricks are estimated to require about 7 hours.

Why is the relation logarithmic and not exponential, as might be expected, is an important question, and interestingly we find the most suitable explanation in the analogous system of DNA self-assembly. The folding of DNA origami exhibits cooperativity at multiple levels, meaning that once a substructure has assembled, it facilitates the assembly of subsequent structures (21; also Shapiro, Bachelet et al, manuscript under review). Here, too, once a subgroup of bricks has been properly assembled, it can serve as a spatial scaffold that increases the probability for subsequent bricks to assemble.

Two issues were critical for efficient assembly: the concentration of the bricks and brick copy number.

Concentration was defined as the ratio of brick pixel area to total pixel area, using area as an approximation for volume. We found that the optimal concentration is between 0.06_C_ to 0.5_C_. Moreover, as long as this concentration is maintained, the system will assemble efficiently. At higher concentrations, efficiency is reduced because of the steric effects of clutter, while at lower concentrations the efficiency is limited by collision probability.

The impact of brick copy number depends on the number of bricks comprising the system, with the highest impact exhibited with large numbers of bricks. For example, at 18 bricks the presence of 2 copies of each brick drastically increased the assembly efficiency. We expect copy number to be critical in more complex systems.
